# Intrathecal Methotrexate-Induced Posterior Reversible Encephalopathy Syndrome (PRES)

**DOI:** 10.4274/Tjh.2012.0191

**Published:** 2014-03-05

**Authors:** Tülay Güler, Özden Yener Çakmak, Selami Koçak Toprak, Seda Kibaroğlu, Ufuk Can

**Affiliations:** 1 Başkent University School of Medicine, Department of Neurology, Ankara, Turkey; 2 Başkent University School of Medicine, Department of Hematology, Ankara, Turkey

**Keywords:** Posterior reversible encephalopathy syndrome (PRES), Methotrexate, magnetic resonance imaging, Fluid-attenuated inversion recovery

Posterior reversible encephalopathy syndrome (PRES) is an acute neuroradiological diagnosis presenting with headache, vomiting, seizure, abnormalities of the mental status, and visual disturbances associated with a breakdown in cerebral vasculature regulation. It has a unique neuroradiological pattern of symmetrical parietooccipital vasogenic edema [1]. The most common causes of this syndrome are sudden arterial hypertension, preeclampsia, eclampsia, uremia, immunosuppressive drugs, and cancer chemotherapies such as cyclosporine, tacrolimus, L-asparaginase, vincristine, gemcitabine, cytarabine, and cisplatin, typically used in cases of hematopoietic malignancies [2,3,4,5,6,7]. Intrathecal methotrexate-induced PRES in an adult is exceedingly rare [8].

A 43-year-old woman was admitted to gynecology with metrorrhagia. Cervical cancer was diagnosed and radical hysterectomy with lymph node dissection was performed. Final pathology and immunohistochemical analyses revealed B-cell phenotype malign lymphoma, which is consistent with Burkitt lymphoma. A chemotherapy treatment protocol with R-Hyper CVAD, consisting of rituximab, cyclophosphamide, vincristine, adriamycin, and dexamethasone plus 12 mg of intrathecal methotrexate without preservative, was then started. Twelve days after chemotherapy she had severe analgesic-irresponsive headache, nausea, motor agitation, and cooperation failure. Her vital signs and laboratory findings were normal. Cranial computed tomography revealed hypodense areas due to edema in the bilateral cerebral hemispheres, predominantly in the posterior regions. Magnetic resonance imaging (MRI) of the brain showed multiple confluent hyperintense lesions in T2-weighted and fluid-attenuated inverse recovery (FLAIR) sequences (Figure 1a), with no contrast enhancement in T1-weighted sequences. PRES was diagnosed and she was admitted to the intensive care unit (ICU) because of decreased alertness and agitation. Intrathecal methotrexate treatment was discontinued. On the second day in the ICU her blood pressure rose and was then normalized by diltiazem infusion. On the third day in the ICU, myoclonic jerks were seen in all extremities. Levetiracetam was started. Myoclonic symptoms were no longer observed. After treatment she had no neurological symptoms. Three weeks later, cranial MRI showed significantly improved brain lesions (Figure 1b). The 6-month follow-up was uneventful. Informed consent was obtained.

During chemotherapy for hematopoietic malignancies, possible causes of neurological symptoms (cerebrovascular disease, metabolic disturbances, neoplasia, and infections) must be excluded by clinical, biological, and imaging findings. During chemotherapy, various types of anticancer drugs are administered, and it is difficult to identify which drug induces PRES. In our case, intrathecal methotrexate treatment was stopped, and the patient’s symptoms were relieved and did not reoccur while her treatment was continued with other anticancer drugs. 

In treatment, the causal factor must be discontinued. The treatment of overdose of intrathecal methotrexate is dilution and removal from the cerebrospinal fluid with specific antidotal therapy. Leucovorin and anti-inflammatory agents are useful [9]. Although PRES is usually reversible with patient recovery and resolution of the imaging findings, it might be recurrent or result in permanent damage [10,11]. 

## Figures and Tables

**Figure 1a:**
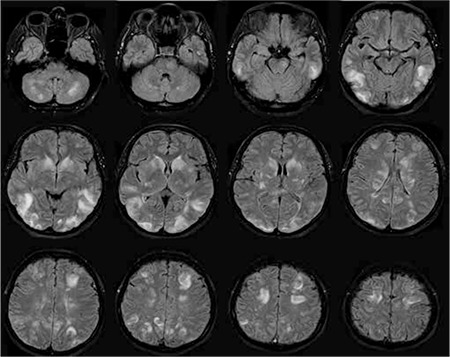
MRI FLAIR images show bilateral multiple subcortical and cortical hyperintense lesions.

**Figure 1b:**
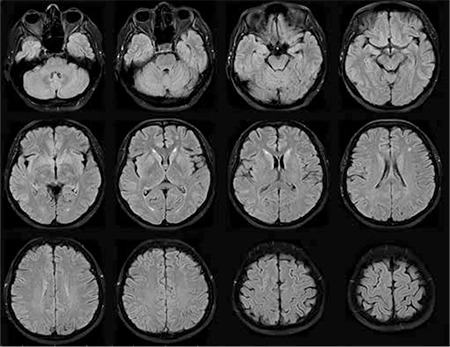
MRI images 3 weeks after developing PRES revealed significant improvement.
